# Clinical characteristics of smokers in an international sample of female mental health service users

**DOI:** 10.1192/j.eurpsy.2026.11952

**Published:** 2026-07-31

**Authors:** M. H. Lo, C. T. L. Chan, M. E. S. Reyes, E. S. Jaya, F. Mukhtar, A. E. Z. Lian, G. Derin, P. D. Bengwasan, G. F. Yuan, A. K. C. Chau, S. K. K. Lam, H. W. Fung

**Affiliations:** 1 Department of Social Work and Social Administration, The University of Hong Kong; 2 School of Nursing, The Hong Kong Polytechnic University, Hong Kong, Hong Kong; 3 Department of Psychology, College of Science, University of Santo Tomas, Manila, Philippines; 4 Faculty of Psychology, Universitas Indonesia, Depok, Indonesia; 5 Department of Psychiatry, Universiti Putra Malaysia, Selangor; 6 Faculty of Social Sciences, Raffles University, Johor, Malaysia; 7 Psychotraumatology and Psychohistory Research Unit, Istanbul University-Cerrahpaşa, İstanbul, Türkiye; 8 Department of Psychology, De La Salle University, Manila, Philippines; 9 School of Education Science, Leshan Normal University, Leshan, China; 10 Department of Psychiatry, The University of Hong Kong; 11 School of Nursing and Health Sciences, Hong Kong Metropolitan University, Hong Kong, Hong Kong

## Abstract

**Introduction:**

Smoking is generally associated with mental health issues, but less is known about the relationship of smoking with different specific psychiatric symptoms in clinical samples across cultures.

**Objectives:**

This study examined the mental health profiles of current smokers in an international sample of female mental health service users.

**Methods:**

We analyzed data from the International Female Mental Health Project, in which 995 female mental health service users from around the world completed an online survey. The survey included the Childhood Trauma Section of the Brief Betrayal Trauma Survey, the Patient Health Questionnaire (PHQ-9), the Somatic Symptom Scale-8 (SSS-8), the International Trauma Questionnaire, the Multiscale Dissociation Inventory, and the Screen for Disordered Eating.

**Results:**

Approximately 12.5% of participants reported themselves as current smokers. Compared to non-smokers, smokers were significantly more likely to report childhood trauma (90.2% vs 96.8%), χ² = 5.69, p = .017, score 15 or above on the PHQ-9 (36.4% vs 50.8%), χ² = 9.55, p = .002, score 13 or above on the SSS-8 (52.7% vs 70.2%), χ² = 13.37, p < .001, screen positive for ICD-11 PTSD/CPTSD (45.8% vs 56.5%), χ² = 4.93, p = .026, and screen positive for dissociative symptoms (39.2% vs 54.0%), χ² = 9.94, p = .002. The two groups did not significantly differ in the rate of probable eating disorders (p = .067).

**Conclusions:**

This study provides updated and cross-cultural data showing that smoking is associated with a wide range of mental health symptoms among female mental health service users. Female mental health service users who smoke have a more severe and complex clinical profiles with more comorbidities, and therefore they may require additional clinical care. Prevention and timely management of smoking in mental health settings are important.

**Disclosure of Interest:**

None Declared

